# Multiple Receptive Field Network (MRF-Net) for Autonomous Underwater Vehicle Fishing Net Detection Using Forward-Looking Sonar Images

**DOI:** 10.3390/s21061933

**Published:** 2021-03-10

**Authors:** Rixia Qin, Xiaohong Zhao, Wenbo Zhu, Qianqian Yang, Bo He, Guangliang Li, Tianhong Yan

**Affiliations:** 1 College of Information Science and Engineering, Ocean University of China, Qingdao 266000, China; qinrixia@stu.ouc.edu.cn (R.Q.); zhaoxh5520@163.com (X.Z.); zwb5437@stu.ouc.edu.cn (W.Z.); Daisy19961001@163.com (Q.Y.); 2 School of Mechanical and Electrical Engineering, China Jiliang University, Hangzhou 310018, China; thyan@163.com

**Keywords:** forward-looking sonar, object detection, underwater fishing net, autonomous underwater vehicle, deep learning

## Abstract

Underwater fishing nets represent a danger faced by autonomous underwater vehicles (AUVs). To avoid irreparable damage to the AUV caused by fishing nets, the AUV needs to be able to identify and locate them autonomously and avoid them in advance. Whether the AUV can avoid fishing nets successfully depends on the accuracy and efficiency of detection. In this paper, we propose an object detection multiple receptive field network (MRF-Net), which is used to recognize and locate fishing nets using forward-looking sonar (FLS) images. The proposed architecture is a center-point-based detector, which uses a novel encoder-decoder structure to extract features and predict the center points and bounding box size. In addition, to reduce the interference of reverberation and speckle noises in the FLS image, we used a series of preprocessing operations to reduce the noises. We trained and tested the network with data collected in the sea using a Gemini 720i multi-beam forward-looking sonar and compared it with state-of-the-art networks for object detection. In order to further prove that our detector can be applied to the actual detection task, we also carried out the experiment of detecting and avoiding fishing nets in real-time in the sea with the embedded single board computer (SBC) module and the NVIDIA Jetson AGX Xavier embedded system of the AUV platform in our lab. The experimental results show that in terms of computational complexity, inference time, and prediction accuracy, MRF-Net is better than state-of-the-art networks. In addition, our fishing net avoidance experiment results indicate that the detection results of MRF-Net can support the accurate operation of the later obstacle avoidance algorithm.

## 1. Introduction

Autonomous underwater vehicles (AUVs) are important tools used in ocean exploration. They can be applied in a wide range of tasks in marine investigation, resource exploration, and military fields. However, underwater fishing nets represent a potentially fatal danger that AUVs often face in complex and unknown marine environments when performing their tasks. Therefore, it is key to develop autonomous recognition of underwater fishing nets to improve the intelligence and autonomous survival ability of AUVs before they are deployed at sea. 

Inspired by the human vision system, object recognition for robots and other intelligent systems is usually conducted with optical sensors. However, due to the refraction and absorption of light by water, the quality of the underwater optical images is usually very poor. Thus, the vision systems of AUVs mainly depend on sonar. Forward-looking sonar (FLS) is one of the main sensors that AUVs use to detect underwater objects. The detected objects can be visualized as forward-looking sonar images. In this way, AUVs can recognize and detect objects from these sonar images and perform various marine tasks, such as path planning [1], underwater archaeology [2,3], and fish identification [4]. Therefore, we can allow the AUV to detect and locate underwater fishing nets with FLS images, thereby avoiding the fishing net with detection results before causing any irreparable damage to the AUV. 

However, because of the complex and changeable characteristics of seawater and the reverberation at the bottom and surface of the sea, FLS images usually have three properties: (1) low resolution, (2) lack of information due to the low gray level of targets, and (3) serious reverberation and speckle noise. These pose great challenges to object detection with FLS images. Researchers have proposed various preprocessing methods to suppress noise interference in image and object detection algorithms based on object proposals [3,5,6,7]. Although these algorithms have made great achievements, the process of choosing object proposals is complex and time-consuming. Moreover, there is still much room to improve the detection accuracy of objects, especially for sonar images collected in the real application scenario of the sea. 

Due to the unique characteristics of FLS images, the research on FLS image detection is not as rich as that of optical images, but some effective works have been developed on object detection of FLS images. In the early research, the appearance characteristics (area, centroid, perimeter, etc.) of the object through the threshold segmentation of FLS images were estimated [8], or complex manual features to extract information from images were proposed [3]. These methods usually have a high number of false positives and complex calculation processes. After AlexNet [9] achieved great successes in the task of ImageNet classification, feature extraction based on deep learning gradually replaced those with manual features that have poor expression abilities. Researchers use the convolutional neural network to extract features from FLS images, and they use these features for classification or detection. Some object detection algorithms for FLS images first obtain a large number of proposals, and then achieve object detection by training these proposals. Some studies have applied the existing object detection algorithms verified on public optical datasets to FLS images and utilized them on AUVs. At present, the object detection algorithms used for FLS images are mostly based on the actual task, and the appropriate algorithm is selected according to the task requirements.

In this paper, we focus on the practical use of the object detection algorithm to recognize fishing nets for AUVs in real-time, and this needs a trade-off between the detecting speed and accuracy of the algorithm. To this end, we propose a multiple receptive field network (MRF-Net). Specifically, in MRF-Net, inspired by the CenterNet [10], we propose an anchor-free approach, where the input images are fed into a fully convolutional network that generates a center-point heatmap which is used to predict object centers. The center-point detector is simpler and more accurate than corresponding anchor-based detectors. Moreover, to extract the features of FLS images faster and better, in MRF-Net, we propose a novel backbone network, which has depth-wise convolution and a multi-branch block using dilated convolution. The number of parameters of depth-wise convolution is reduced significantly in our architecture. The multi-branch block provides the multi-scale of receptive fields. Moreover, we use a combination layer of instance normalization (IN) and batch normalization (BN) to improve the generalization performance of the proposed network without extra computation. In addition, to improve the detection effect of targets, we introduce mixup [11], which can increase the diversity and numbers of targets. We trained and tested our network on data collected in the sea by comparing it with some of the most popular object detection algorithms. To provide the network with the ability to distinguish fishing nets from other entanglements, we added two other entanglements (cloth and plastic bags) to the collected data. We also evaluated our network in the sea by detecting fishing nets in real-time with the NVIDIA Jetson AGX Xavier of our AUV platform. The experimental results show that in terms of computational complexity, inference time, and prediction accuracy, MRF-Net is better than state-of-the-art networks. In addition, our results indicate that the detection results of MRF-Net can support the accurate operation of the later obstacle avoidance algorithm in real-time.

The remainder of this paper is organized as follows: we introduce related works on object detection in Section 2; in Section 3, the network architecture and relevant formulation are described; in Section 4, we introduce our datasets, the implementation details, and training of the network; Section 5 shows the experimental results and analysis via an evaluation of our network on an FLS images dataset in comparison with state-of-the-art networks, and we also report the real-time detection of fishing nets with our network in the sea in the section; finally, we give our conclusions in Section 6.

## 2. Related Work

**Figure 1:**
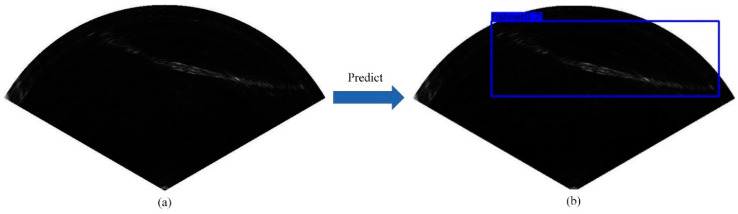
An example of object detection. (**a**) A test forward-looking sonar (FLS) image with a fishing net; (**b**) the predicted position of the fishing net in the image (blue box).

Object detection is an active field in computer vision, including predicting the category of objects in the image and marking the position of objects with the bounding boxes. Figure 1 is an example of object detection with the aim of predicting the categories and positions of the objects in the image.

Early object detection algorithms were mostly based on manual features such as histogram of oriented gradient (HOG) [12] and scale invariant feature transform (SIFT) [13]. The defects of manual features in the feature expression ability have a largely negative effect on detecting accuracy. Therefore, increasing efforts are being made to designing algorithms that learn features autonomously, such as the deep convolution neural network (DCNN). One of the advantages of DCNN is that its multi-layer convolutional structure can autonomously learn very robust features with a good expression ability. Researchers have proposed many outstanding object detection algorithms based on DCNN. Currently, the popular object detection algorithms can be roughly divided into two categories: the two-stage detector and the one-stage detector. 

Two-stage detectors generate box proposals that are relevant to object positions and then classify and regress these proposals. The regions with convolutional neural network (R-CNN) [14] is one of the first successful two-stage detectors, which uses selective search to produce object proposals and classifies these object proposals through AlexNet [9]. Compared with the traditional methods, R-CNN has achieved remarkable performance in prediction accuracy and created an era of deep learning for object detection algorithms. In order to reduce the calculation and speed up the detection, Fast R-CNN [15] was proposed, using only one convolution neural network (CNN) to extract features of proposals with region of interest (RoI) pooling. Faster R-CNN [16] was proposed, using the region proposal network (RPN) to generate region proposals instead of selective search, which reduced the time to generate region proposals. Besides, compared with Fast R-CNN [15], Faster R-CNN [16] achieves real end-to-end training. Moreover, many effective novel networks have been proposed to further improve detection accuracy, such as region-based fully convolutional network (R-FCN) [17], feature pyramid network (FPN) [18], and Mask R-CNN [19]. These architectures improve the detection accuracy but have a slow detection speed, so some literatures propose the one-stage detectors with a real-time detection speed. 

One-stage detectors classify and regress anchors directly without specifying their content. You only look once (YOLO) [20] is the first one-stage object detection algorithm, which takes the whole image as the input of the network and directly regresses the positions and categories of the bounding boxes in the output layer. Although YOLO has a very fast detection speed, its positioning accuracy is greatly reduced when compared with that of Faster R-CNN. Its descendants [21,22] display an updated framework and improved accuracy while maintaining the detection speed. The single shot multibox detector (SSD) [23] performs multi-scale detection and bounding box regression on multi-scale feature maps, which alleviates the problem of missing detection of small targets in YOLO and maintains a fast detection speed. Furthermore, more advanced one-stage detectors, such as RetinaNet [24] and receptive field block (RFB) Net [25], use deeper CNNs as backbones and apply certain techniques, such as dilated convolution [26] and focal loss [24], whose accuracy is comparable and even superior to that of state-of-the-art two-stage methods. However, the more prediction accuracy improves, the more time needed for detection. 

In addition, despite the continuous developments and improvements in object detection, most are aimed at optical image datasets. Due to the complex and changeable noises of FLS images, many studies put forward different algorithms for different application scenarios to detect objects in FLS images. However, there is no universal object detection algorithm for FLS images at present.

Petillot et al. [8] segment the forward-looking sonar image with adaptive threshold technology; extract the features from the segmented obstacle regions, such as area, perimeter, and moments; and track obstacles for AUV obstacle avoidance and path planning using a Kalman filter. Weng et al. [27] enhance the image contrast before using the improved Otsu threshold in order to distinguish the foreground from the background in FLS images, and they use a contour detection algorithm to identify the contour of the segmented image. The object position is further calculated according to the contour of the object. Hurtos et al. [28] use template matching to detect underwater chain links and perform normalized cross-correlation between the input image and a set of templates. The object is in the position that produces maximum correlation. In addition, convolutional neural networks have also been used for detecting objects in FLS images [29], where a CNN is trained on box proposals generated by a sliding window and screened with intersection over union (IoU). This method has a high recall but produces a large number of false positives. An end-to-end system [30] was built for object detection and recognition, which improves false positives by explicitly modeling the detection process as proposals. Another method [6] was proposed to regress a score related to objects directly from a FLS image, which produces a high recall with only few proposals per image. Neves et al. [3] propose a rotation-invariant method to detect and identify underwater objects, which uses HOG as feature extractor and combines a multi-scale oriented detector with a support vector machine to recognize trained objects. However, most of these algorithms use traditional feature extraction methods which cannot extract features with strong representation ability. In addition, some algorithms use CNN to extract better features, but the detection time increases because of the sliding window used to extract proposals. Zacchini et al. [31] realize the recognition and location of potential objects in FLS images through the existing Mask R-CNN, and achieve good accuracy and recall, but the inference time of Mask R-CNN is about 200 ms, which is not suitable for real-time detection. By comparing the performance of several different object detection algorithms on the same FLS image dataset, Kvasic et al. [32] find a robust and reliable object detection network for the detection and tracking of human divers. In summary, due to the different task targets, most of the studies on FLS image object detection explore a suitable algorithm from the perspective of specific problems and the actual situation. In this paper, we propose the use of MRF-Net to detect underwater fishnets, as it can learn the more robust features of FLS images autonomously with much less time than other methods.

## 3. Method

### 3.1. Data Preprocessing

**Figure 2:**
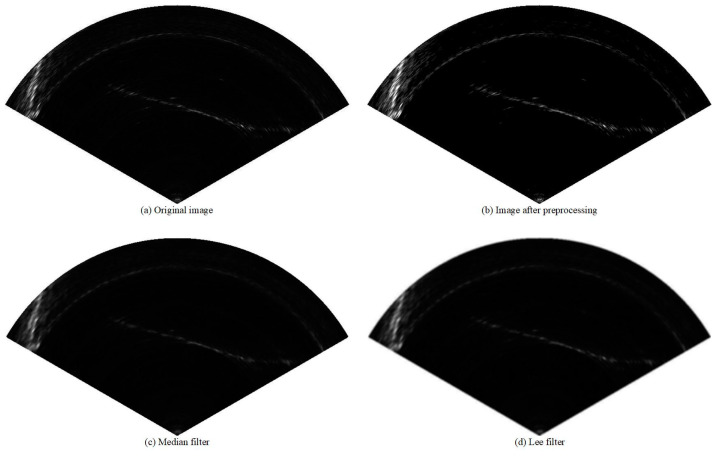
An example of various preprocessing methods: (**a**) the original image; (**b**) the preprocessed image with our method; (**c**) the preprocessed image with median filter; (**d**) the preprocessed image with Lee filter.

As described in the Section 1, there are usually a considerable number of reverberations and speckle noises in FLS images. At present, there is no effective general denoising method to filter the noise in FLS images. Most studies choose the denoising method according to their actual task. The common denoising methods include median filtering and Lee filtering. In our task, we found that denoising algorithms usually inevitably cause images to blur, which affects the detection results. Therefore, we preprocessed the images with threshold segmentation to reduce the interference of noises on the objects. Because the gray levels of the target and noise are similar, we used the gray stretching operation to improve the contrast between the target and the noise before threshold segmentation. This solves the problem of missing targets due to threshold segmentation. Equations (1) and (2) represent the compute mode of gray stretch and threshold segmentation, respectively:

(1) $$GS_{dst}\left(x,y\right)=\{\begin{array}{l} a*GS_{src}\left(x,y\right)+b,\;\;if<255,\\ 255,\;\;\;\;\;\;\;\;\;\;\;\;\;\;\;\;\;\;\;\;\;\;\;\;\;\;\;\;otherwise. \end{array}$$

where $GS_{src}\left(x,y\right)$ is the pixel value at (*x*, *y*) before gray stretching, $GS_{dst}\left(x,y\right)$ is the pixel value at (*x*, *y*) after gray stretching, and the upper limit of $GS_{dst}\left(x,y\right)$ is 255. Parameters a and b are used to control the degree of stretching. After various attempts, we set *a* = 1.5, *b* = 0 in our experiments:

(2) $$TS_{dst}\left(x,y\right)=\{\begin{array}{l} TS_{src}\left(x,y\right),\;\;TS_{src}\left(x,y\right)>thresh,\\ 0,\;\;\;\;\;\;\;\;\;\;\;\;\;\;\;\;\;\;otherwise. \end{array}$$

where $TS_{src}\left(x,y\right)$ is the pixel value at (*x*, *y*) before threshold segmentation, $TS_{dst}\left(x,y\right)$ is the pixel value at (*x*, *y*) in the segmented image, and thresh is the threshold that equals the pixel mean value of the sector area. The results after preprocessing are shown in Figure 2. The influence of each filtering algorithm on object detection is described in detail in the Ablation Experiment described in Section 5.3.

### 3.2. Network Architecture

**Figure 3:**
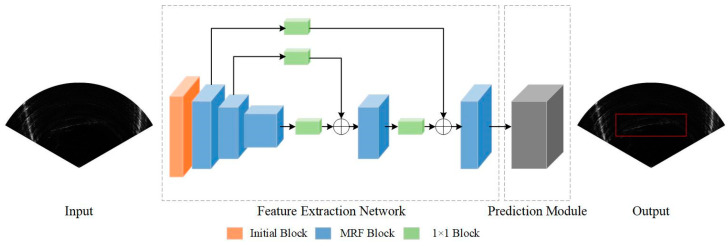
An illustration of multiple receptive field network (MRF-Net) architecture.

In this section, we introduce the structure of the proposed MRF-Net in detail. Its structure is mainly divided into two parts: the feature extraction network, which is mainly responsible for fusing different levels of features from FLS images, and the prediction module, which is responsible for locating the boundary box of the object. Our network architecture is shown in Figure 3.

#### 3.2.1. Feature Extraction Network

The feature extraction network is composed of the initial block and an encoder-decoder module, which is made by stacking the MRF block in a sequential manner. Different from the classification, object detection needs not only high-resolution feature maps but also large receptive fields. Therefore, we propose using an encoder-decoder module to ensure the production of a high-resolution feature map and an MRF block to increase the high-level receptive field. 

**Figure 4:**
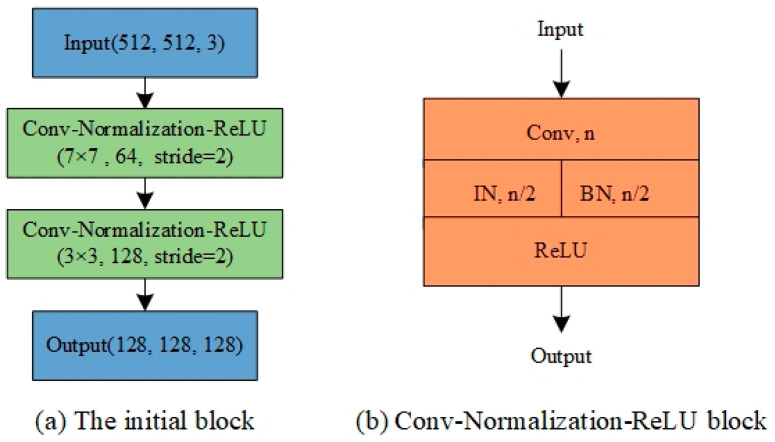
The initial block of our proposed network. (**a**) Overall structure of the initial block, in which the size, number, and step size of the convolution kernel are annotated. (**b**) The specific structure of Conv-Normalization-ReLU block, *n* is the number of convolution kernels.

The initial block of our network consists of two Conv-Normalization-ReLU blocks, which has a convolutional layer followed by an IBN layer and a rectified linear unit (ReLU) activation layer. The IBN layer integrates instance normalization (IN) and batch normalization (BN) as building blocks. The details are shown in Figure 4. 

**Figure 5:**
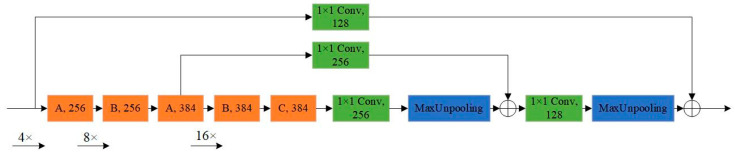
An illustration of the encoder-decoder module. According to different requirements, the MRF block has three different structures (A, B, and C). Note that the numbers in each block represent the number of filters. When the down-sampling factor reaches 16, the resolution of the feature map is not reduced.

The detailed structure of the encoder-decoder module is shown in Figure 5. The encoder module extracts the features through the MRF block, which has three different variants. The decoder module is responsible for up-sampling the features and fusing them with the corresponding features with the same size. Inspired by DetNet [33], we reduced the down-sampling factor of input images to reduce the loss of semantic features of small object at a high-level. The down-sampling factor of the final output feature of the encoder block is set to 16.

**Figure 6:**
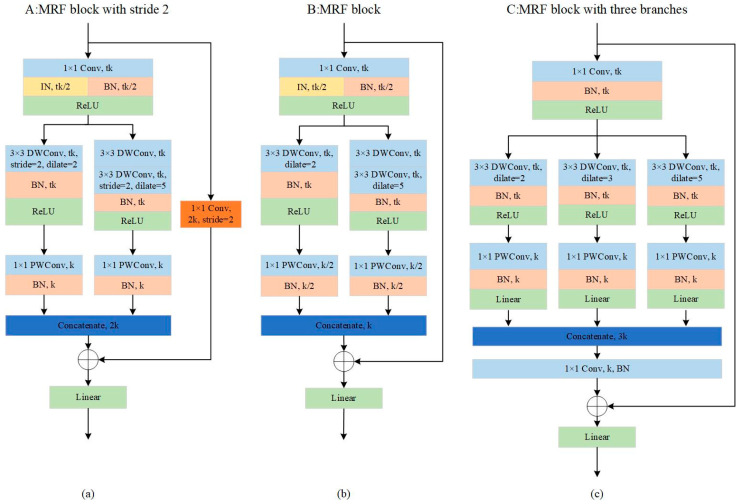
The MRF block, where k is the number of input channels, and t is the expansion factor to increase channels. (**a**) The MRF block with stride 2, which reduces the size of feature map by half and doubles the number of channels; (**b**) the MRF block; (**c**) the MRF block with three branches that have different dilated rates.

As the decrease in the down-sampling factor leads to the decrease in the receptive field, which is not conducive to locating objects in FLS images, we proposed the MRF block based on the inverted residual block [34] and added the dilated convolution to increase the receptive field. The specific structure is shown in Figure 6. In the MRF block, we obtain different scales of receptive fields through the multi-branch structure with different dilated rates. Multi-scale receptive fields are more conducive to learning multi-scale objects. Compared with other network structures, MRF-Net uses wider blocks to achieve real-time and accurate object detection with fewer layers. 

As shown in Figure 6b, a 1 × 1 convolution layer called the “expansion” layer is used to expand the input channels, which is helpful for the next convolution layer to obtain more rich features. Following this, different scale depth-wise (DW) convolution and a 1 × 1 point-wise (PW) convolution, also called depth-wise separable convolution, occur in the two branches. DW convolution has the same number of kernels as that of the previous layer. Each only performs the convolution operation with the corresponding channel. The advantage of this operation is that it greatly reduces the number of parameters and the operation cost. However, it is not conducive to exchanging the information between channels, and it does not effectively use the feature information of different channels in the same space. Therefore, PW convolution is needed to combine these feature maps to generate a new feature map. Depth-wise separable convolution is widely used in lightweight networks [34,35,36]. In addition to depth-wise separable convolution, we also used some other techniques to optimize the network and improve the accuracy of detection.

**Figure 7:**
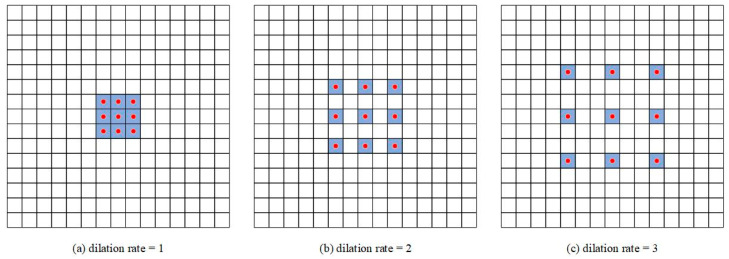
An illustration of dilated convolution. The positions of red dot are the actual positions that accomplish convolution operation with kernels. (**a**) A 3 × 3 convolution kernel with dilation rate 1. (**b**) A 3 × 3 convolution kernel with dilation rate 2. (**c**) A 3 × 3 convolution kernel with dilation rate 3.

- **Dilated Convolution.** This introduces a new parameter, dilation rate, into standard convolution. The actual positions where kernels implement the convolutional operation vary with the dilation rate, as shown in Figure 7. The purpose of dilated convolution is to replace the maximum pooling that cause information loss and provide a larger receptive field when the amount of computation is equivalent. However, dilated convolution causes the discontinuity of the receptive field, that is, the gridding issue. Thus, when using dilated convolution with the same dilation rate, many neighboring pixels will be ignored and only a small part will be calculated. Moreover, with the increase in dilation rate, the local information, especially the local information of the center point, is seriously ignored. Therefore, we combined the dilated convolutions with different dilation rates to detect the large and small targets correctly at the same time. As shown in Figure 6a,b, one branch has a 3 × 3 convolution layer with a dilation rate of 2, and another branch has two 3 × 3 convolution layer with dilation rates of 1 and 5, respectively. After the down-sampling coefficient reaches 16, we used the three-branch MRF block, setting the dilation rate to 2, 3, and 5, respectively, as shown in Figure 6c.
- **IBN Layer.** We used IBN-Net [37] for reference to add the IBN layer to the MRF block, which combines instance normalization (IN) and batch normalization (BN) reasonably to improve the learning ability and generalization ability of the network. IN learns features that are invariant to appearance changes but reduces the useful information about content of images, while BN is essential for preserving content related information. It is known that the low-level features of CNN reflect appearance differences, and the high-level features reflect semantic information. Therefore, we used this property by introducing IN to the MRF block. As shown in Figure 6, we placed IN only at the lower layer, which can filter the information that reflects the appearance and retain the semantic information at the same time. Moreover, in order to retain the content information of the image in the lower layer, we set half of the normalization features to IN, and the other half to BN. To retain the semantic information, we only added IN to low layers whose downsampling factor was less than 16.
- **Activation Function.** The nonlinear function is usually used as the activation function to add nonlinearity to the CNN and enhance the ability of feature expression. ReLU is widely used as the nonlinear activation function. Compared with sigmoid and other functions, it has no gradient vanishing problem and is easy to calculate. However, it is found that it is easy to lose information when the ReLU operation is performed on low dimensional features [34]. To solve the problem of information loss, we used the linear activation function to replace ReLU after the DW convolution layer as shown in Figure 6.

#### 3.2.2. Prediction Module

**Figure 8:**
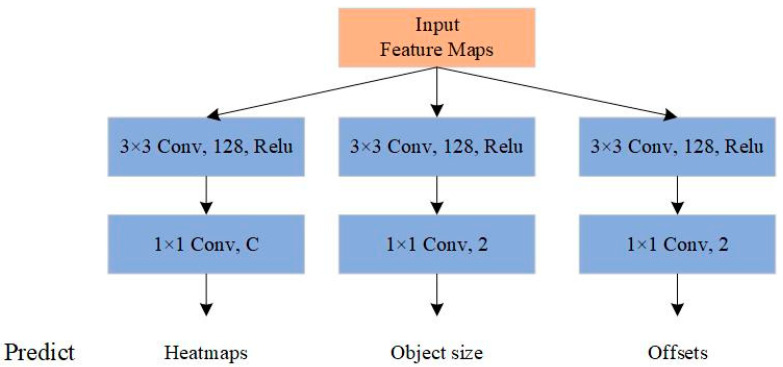
The structure of the prediction module. C is the number of key point types. Using the predictions, we can locate the center point and estimate the size of objects.

The feature extraction network is followed by a prediction module, which is used to predict the center point and size of objects. The structure of the prediction module is illustrated in Figure 8. 

As shown in Figure 8, the prediction module has three branches: one branch is responsible for generating the heatmap with C output channels that can predict the position of the center point of the objects in FLS image, where C is the number of classes in the dataset; the second branch with 2 output channels is in charge of predicting the width and the height of objects; the third branch also has 2 output channels to predict the local offsets for each center point. After the center point, the corresponding width and height of objects are predicted, and we can obtain the boundary boxes of the predicted objects.

## 4. Implementation and Experimental Details

**Figure 9:**
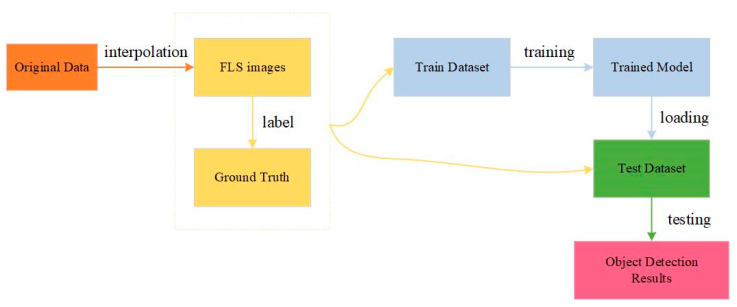
An illustration of training and testing of networks in our experiment.

In this section, we present the details of our datasets and network training for FLS images. The entire experimental procedure of FLS images detection is shown in Figure 9. We obtain the original data matrix with forward-looking sonar. A FLS image is generated by interpolation from the data matrix.

### 4.1. Dataset

The FLS images used in our experiment were collected with a multi-beam forward-looking sonar—Gemini 720i—at the wharf of the Qingdao Scientific Investigation Center. The relevant technical parameters of the Gemini 720i are listed in Table 1. 

**Figure 10:**
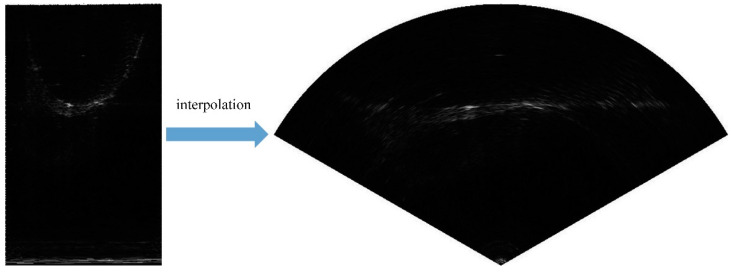
The comparison between image before interpolation (**left**) and image after interpolation (**right**).

According to the scanning principle of forward-looking sonar, each received beam of Gemini 720i returns one column of data, and each frame of image obtains 256 columns of data. However, because of the inconsistency of the coordinate system, if they are directly spliced into an image, the objects in the image will be deformed, and the accurate orientation of the objects cannot be obtained, which will have a negative impact on the AUV in completing the detection and obstacle avoidance task. Therefore, we used the interpolation algorithm based on bilinear interpolation to interpolate the image and finally obtained the complete sonar forward-looking image. The results after interpolation are shown in Figure 10b.

**Figure 11:**
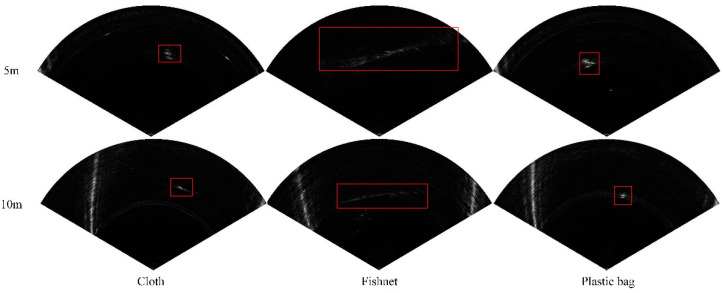
Some examples of the ground-truth in the dataset.

Accurate ground truth of images is very important for supervised object detection of FLS images. In order to obtain more accurate labels, we used the pixel level image annotation tool LabelMe developed by the MIT to mark the FLS images manually and obtain the ground truth used for object detection as shown in Figure 11. In order to enable the model to distinguish between the fishing net and other obstacles, we added the cloth and plastic bag to the dataset. Our dataset includes three kinds of obstacles at different distances (0–5 and 5–10 m): fishing net, cloth, and plastic bag. The proportion of datasets in each category is about 1:1:1. We randomly selected 80% of the images as the training set and the remaining 20% as the testing set. Additionally, we randomly selected about 20% images as the validation set in the training set. More precisely, the dataset consists of 10,995 training images, 3667 validation images, and 3670 testing images.

### 4.2. Loss

We denoted an input image as $X\in R^{W\times H\times 3}$ with width W and height H. When an image was fed into the network, we obtained three output maps: a center point heatmap $\hat{M}\in {\left[0,\;1\right]}^{\frac{W}{D}\times \frac{H}{D}\times C}$, a regression of object size $\hat{S}\in R^{\frac{W}{D}\times \frac{H}{D}\times 2}$, and a prediction of local offsets $\hat{O}\in R^{\frac{W}{D}\times \frac{H}{D}\times 2}$. We set the output stride of downsampling D=4 and object categories of dataset C=3 in the literature.

For an object in the sonar image, there is one ground-truth positive location at the center point $p^{\left(i\right)}=\left(\frac{x_{1}^{\left(i\right)}+x_{2}^{\left(i\right)}}{2},\frac{y_{1}^{\left(i\right)}+y_{2}^{\left(i\right)}}{2}\right)$ generated by a ground-truth box $\left(x_{1}^{\left(i\right)},\;y_{1}^{\left(i\right)},x_{2}^{\left(i\right)},y_{2}^{\left(i\right)}\right)$. However, a false center-point detection closed to the ground-truth point can also generate a bounding box that has a sufficient overlap with the ground-truth box. Therefore, we reduce the penalty on negative positions within a certain radius of the positive position. As in CornerNet [36], we used Gaussian distribution to represent penalty reduction. After down-sampling in the network, we computed $\tilde{p}=\left(\lfloor \frac{p_{x}}{D}\rfloor ,\lfloor \frac{p_{y}}{D}\rfloor \right)$ to replace p in the output heatmap. We denoted the ground-truth of the center-point heatmap at location (x,y) for class c as $M_{xyc}=\exp\left(-\frac{{\left(x-{\tilde{p}}_{x}\right)}^{2}+{\left(y-{\tilde{p}}_{y}\right)}^{2}}{2\sigma_{p}^{2}}\right)$, where $\sigma_{p}$ is the adaptive radius according to object size [10,38]. We defined the predicted score at location (x,y) for class c in the center-point heatmap as ${\hat{M}}_{xyc}$. The loss between the prediction ${\hat{M}}_{xyc}$ and ground-truth $M_{xyc}$ is a logistic regression with the focal loss [24]:

(3) $$L_{center}=-\frac{1}{N}\overset{W}{\underset{x=1}{\sum }}\overset{H}{\underset{y=1}{\sum }}\overset{C}{\underset{c=1}{\sum }}\{\begin{array}{c} {\left(1-{\hat{M}}_{xyc}\right)}^{\alpha }\log\left({\hat{M}}_{xyc}\right),\;\;\;\;\;\;\;\;\;\;\;\;\;\;\;\;\;\;\;\;\;\;\;\;\;if\;M_{xyc}=1,\\ {\left(1-M_{xyc}\right)}^{\beta }{\left({\hat{M}}_{xyc}\right)}^{\alpha }\log\left(1-{\hat{M}}_{xyc}\right),\;otherwise. \end{array}$$

where α and β are the hyper-parameters of focal loss [24] and N is the number of center points in FLS image X. According to [36], we set α=2 and β=4 in our experiments. 

In addition, we denoted the ground truth of object size for each center-point i as $s^{\left(i\right)}=\left(x_{2}^{\left(i\right)}-x_{1}^{\left(i\right)},y_{2}^{\left(i\right)}-y_{1}^{\left(i\right)}\right)$ and the prediction size for all object types at each center point as $\hat{S}\in R^{\frac{W}{D}\times \frac{H}{D}\times 2}$, which reduced the calculation costs. We used the $L_{1}$ loss to compute the object size loss:

(4) $$L_{size}=\frac{1}{N}\overset{N}{\underset{i=1}{\sum }}\left|{\hat{S}}_{{\tilde{p}}^{\left(i\right)}}-s^{\left(i\right)}\right|.$$

Because we applied down-sampling many times in the network to reduce computation and obtain global information, the size of output was smaller than the input. Therefore, a pixel located at (x,y) in an input image was mapped to the pixel located at $\left(\lfloor \frac{x}{D}\rfloor ,\lfloor \frac{y}{D}\rfloor \right)$ in the output heatmap. However, when the output heatmap was remapped back to the input image, some errors inevitably occurred, causing inaccurate detection of small objects. To eliminate the errors, we predicted the local offsets $\hat{O}\in R^{\frac{W}{D}\times \frac{H}{D}\times 2}$ to adjust the location of the predicted center point before remapping the input images. Since we only focus on the location of the center point, we only calculated the offset loss of the center point and ignored the loss of other locations. The ground truth of local offsets is shown in the following formula:

(5) $$o_{p}=\left(p_{x}-\lfloor \frac{p_{x}}{D}\rfloor ,p_{y}-\lfloor \frac{p_{y}}{D}\rfloor \right).$$

We used the $L_{1}$ loss to compute the offset loss:

(6) $$L_{offset}=\frac{1}{N}\sum_{i=1}^{N}\left|{\hat{O}}_{{\tilde{p}}^{\left(i\right)}}-o^{\left(i\right)}\right|,$$

where $o^{\left(i\right)}$ is the ground-truth offsets for center point i, and ${\hat{O}}_{{\tilde{p}}^{\left(i\right)}}$ is the corresponding predicted offset. 

In summary, the allover loss in the training is:

(7) $$L=L_{center}+\lambda_{size}L_{size}+\lambda_{offset}L_{offset},$$

where $\lambda_{size}$ and $\lambda_{offset}$ are the weights of the size and offset, respectively. We set $\lambda_{size}=0.1$ and $\lambda_{offset}=1$ in our experiments.

### 4.3. Training Details

In this subsection, we introduce the details of training, including training parameters and some training techniques that were used to improve the performance of the model.

#### 4.3.1. Mixup Strategy

Mixup [11,39] was proposed as a simple and effective data augmentation method to reduce generalization errors and alleviate the sensitivity of the adversarial samples in the classification network. It uses virtual samples for training, which mix up two samples selected randomly at a certain mixing ratio. The mixed labels corresponding to the virtual samples are generated using the same ratio at the same time. The relevant formula is as follows:

(8) $$\{\begin{array}{c} \tilde{x}=\lambda x_{i}+\left(1-\lambda \right)x_{j}\\ \tilde{y}=\lambda y_{i}+\left(1-\lambda \right)y_{j} \end{array},$$

where $\left(x_{i},y_{i}\right)$ and $\left(x_{j},\;y_{j}\right)$ are two different sample-labeled pairs selected randomly from the training dataset, and λ∈[0, 1] is the blending ratio whose distribution is drawn from a beta distribution B(α,β). The hyper-parameters α and β control the mixing degree between sample-labeled pairs.

**Figure 12:**
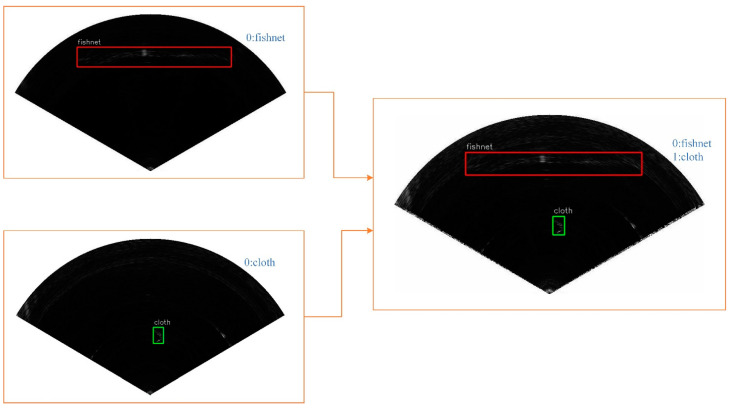
Mixed images for object detection. The images are mixed by pixel, and the bounding box of objects are also merged into the mixed image.

To increase the accuracy of the network, we used the mixup method in our training process. An example of mixup for object detection with a high mixing ratio is shown in Figure 12. Inspired by [11], we found that increasing the mixing ratio can make the object more active in the mixed image, which is conducive to object detection. In our experiment, we chose a beta distribution B(0.5,0.5) as the blending ratio.

#### 4.3.2. Training Parameters

We implemented our MRF-Net detector with the Pytorch framework. The network was initialized randomly before training without using the model trained with the classification dataset for pretraining. We set the biases in the convolution layer which predicts the center point heatmap according to [24]. During training, we added the FLS images with a resolution of 512 × 512 into the network and obtained the output feature maps with the resolution of 128 × 128. Our training strategy of data augmentation follows CenterNet [10]. We used the optimization strategy of stochastic gradient descent (SGD) to gradually reduce the loss and improve the accuracy of target detection until the training loss converged. The settings of hyper-parameters are as follows: mini-batch size, 16; momentum, 0.9; weight decay, 0.0005. 

For the learning rate, we used the warmup strategy to avoid gradient explosion in the initial training stage. We increased the learning rate from 10^−6^ to 10^−3^ in the first five epochs. Then, we used a cosine decay strategy [40] to adjust the learning rate. The specific formula is as follows:

(9) $$l_{n}=l_{initial}\times 0.5\times \left(1+\cos\left(\frac{n}{N_{epoch}}\pi \right)\right),$$

where $n\;(5<n\leq N_{epoch})$ is the current number of epochs, $l_{initial}$ is the initial learning rate of $10^{-3}$ and $N_{epoch}=200$ represents the total number of epochs.

## 5. Experimental Results and Analysis

The network training of our experiments was run on an NVIDIA Quadro M5000 card, and the well-trained model was then tested on an NVIDIA Jetson AGX Xavier embedded system module. Our network allows any size of image as input. The image is scaled to 512 × 512 in the data preprocessing stage, and then fed into the network for feature extraction. We compared our network with the existing popular object detection algorithms on different indicators. The settings of these compared networks are the same as those in the original papers. Specifically, in Section 5.1, we evaluate the accuracy of MRF-Net and show that the performance of our network can reach or even surpass that of the most advanced algorithms in terms of prediction accuracy. In Section 5.2, we verify the detection speed of our network, and prove that it can meet the real-time requirements and is faster than other networks. In Section 5.3, we conduct ablation research to prove the influence of each component of MRF-Net on the detection results and identify the reason why MRF-Net is superior to other algorithms. In Section 5.4, we further test our network by detecting fishing nets in real-time in the sea with the NVIDIA Jetson AGX Xavier embedded system module of an AUV platform in our lab.

### 5.1. Accuracy

Our dataset consists of three categories of obstacles: cloth, fishnet, and plastic bag. Compared with fishing net, the area of cloth and plastic bag is smaller, which belongs to small objects. Moreover, due to the different materials, the FLS images of the cloth are weaker than those of the plastic bag, which makes the detection more difficult. 

**Figure 13:**
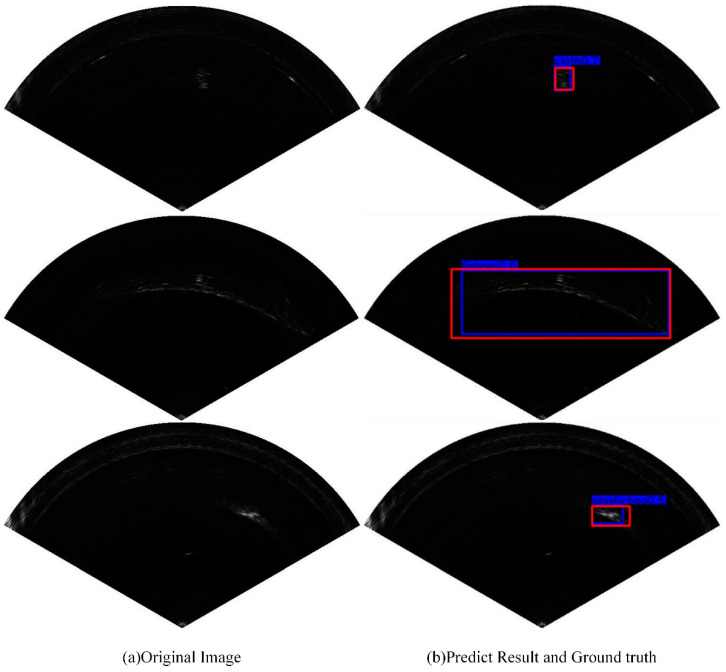
The detection results of FLS images. (**a**) Original FLS images with different objects. (**b**) Detection results of MRF-Net with blue boxes and ground truth with red boxes.

Figure 13 shows the object detection results of our network on the collected FLS image dataset. The bounding boxes in red in Figure 13b represent the ground truth, and the bounding boxes in blue are the corresponding detection results. From Figure 13, we can observe that the proposed MRF-Net has an outstanding detection performance compared with that of ground truth. In order to evaluate our network more objectively, we compared our network with some state-of-the-art object detection algorithms using our collected FLS dataset in the sea in terms of the mAP (mean average precision) and the AP (average precision) of each category. 

The AP of a certain category is the area under the precision-recall curve (P-R curve). To calculate the precision and recall, we need to determine true positives (TP), false positives (FP), true negatives (TN), and false negatives (FN). The definitions of these indicators are shown in Table 2. In this paper, the decision threshold is 0.5. That is to say, a predicted bounding box is positive if its IoU with ground truth is higher than 0.5.

Precision refers to the probability that the predicted bounding boxes are determined to be positive examples and are indeed positive examples. Its formula is defined as follows:

(10) $$P\;\left(Precision\right)=\frac{TP}{TP+FP}$$

Recall refers to the proportion of the predicted bounding boxes correctly classified as positive examples to all ground truth:

(11) $$R\left(Recall\right)=\frac{TP}{TP+FN}$$

Because the specific expression of the P-R curve is difficult to obtain, we used the estimation method in COCO API, which means we calculated the corresponding *P* when *R* = 0.00, 0.01, 0.02, ..., 1.00 respectively. Then AP of a certain category is the average value of these P values. mAP is the average of AP of all categories:

(12) $$mAP=\;\frac{1}{C}\overset{C}{\underset{c=1}{\sum }}AP_{c},$$

where *C* is the number of object categories. 

Table 3 shows the prediction results of our network, compared to CenterNet-dla [10], Faster RCNN [16], YoloV3 [21], SSD300 [23] and RFBNet [25] in terms of the mAP and AP of each category. From Table 3 we can see that, in terms of average precision of predicting the cloth, MRF-Net is much better than all other networks. As for predicting plastic bag, the performance of MRF-Net is comparable to that of RFBNet and CenterNet-dla, all of which are better than the other three networks. This indicates that our proposed network performs well in detecting small and weak objects. As for predicting fishing net, the performance of MRF-Net is slightly worse than that of Faster RCNN and CenterNet-dla, but much better than the other three networks. However, in terms of mAP, MRF-Net can achieve 90.3%, which is better than all other networks.

### 5.2. Inference Time

In order to verify the real-time performance of our network, we tested it on the Nvidia Xavier embedded system module of the AUV platform in our lab. We evaluated and compared the performance of our proposed network with the abovementioned detectors in Section 5.1 in terms of number of parameters, model size, the amount of calculation (GFLOPs), and frames per second (FPS). The results are shown in Table 4. From Table 4, we can see that the proposed MRF-Net can process as many as 11 frames per second, and the inference time is 89 ms, which is faster than that of the other networks. Moreover, in terms of the amounts of parameters and the amount of calculations, Table 4 also shows that MRF-Net has the fewest parameters and smallest model size. The GFLOPs of MRF-Net are also much smaller compared to those of the other networks. In summary, the computational complexity of MRF-Net is smaller than other networks. Therefore, MRF-Net is efficient, and it can meet the strict computing and memory requirements of embedded devices and can complete real-time tasks accurately.

### 5.3. Ablation Experiment

In order to better understand the influence of the techniques we used in the proposed MRF-Net on the prediction performance, we conducted ablation experiments for each component. The results are summarized in Table 5. As shown in Table 5, we investigated the effect of the preprocessing method, the IBN layer, dilated convolution and the mixup strategy in MRF-Net. When these components are not added, our architecture can achieve 87.6% mAP; after adding different components, the detection accuracy is improved to varying degrees.

- **Preprocessing Methods.** As described in Section 3.1, we used gray stretching and threshold segmentation to preprocess the FLS images, which can suppress the noise interference to a certain extent, and achieve the effect of highlighting the target. This operation improves the detection performance by 0.6% (from 88.6% to 89.2%). In addition, we can see from the Table 5 that the median filter and Lee filter did not improved the detection accuracy but led to a decrease in different amplitudes.
- **IBN layer.** The IBN layer combines two normalization methods, IN and BN, and it not only retains the features of appearance invariants but also retains rich semantic features. In the experiment, we added the IBN layer to the shallow layer of the network, which improved the learning ability and generalization ability of the network and further improved the mAP by 0.5% (from 89.2% to 89.7%).
- **Dilated Convolution.** Dilated convolution is a method that can increase the receptive field without increasing the number of parameters. We introduced dilated convolution in our architecture to ensure the high-resolution of the feature map without reducing the receptive field. We also combined convolution kernels of different sizes with different dilated rates to achieve accurate detection of multi-scale targets. This is one of the reasons why MRF-Net can perform well in detecting small objects. As described in Table 5, selecting the dilated convolution can improve the accuracy by 1.0% (from 87.6% to 88.6%).
- **Mixup Strategy.** The scale of our own dataset used in our experiment is far less than that of the open source datasets, so there is a risk of over fitting during training. In order to enrich the dataset and reduce the risk of over fitting, we use the mixup strategy which randomly synthesizing virtual data. We used this technique in the training process, which further boosted the performance by 0.6% (from 89.7% to 90.3%) for our MRF-Net.

### 5.4. Real-Time Experimental Results

**Figure 14:**
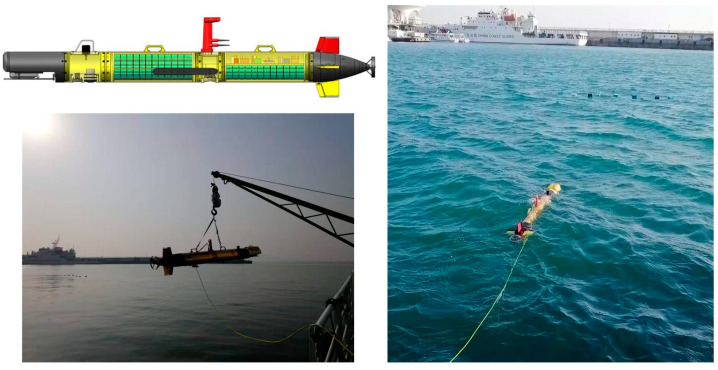
The structure of our autonomous underwater vehicle (AUV) platform and the scenes of the fishing net avoidance experiment.

**Figure 15:**
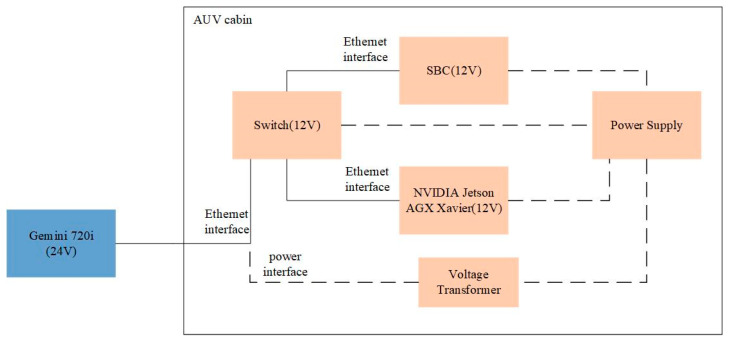
An illustration of the hardware connection between devices in our real-time detection experiment. Note that the dotted line represents the connection between the device and power supply. Since the power supply voltage of Gemini 720i is different from other embedded devices, its power is supplied with a transformer.

In order to further prove that our detector can be applied to the actual detection task, we carried out a real-time detection experiment in the experimental station of the Shandong Academy of Sciences Institute of Marine Instrumentation, which is different from the sea area where we collected the dataset to train the network. Due to the influence of water quality and reverberation, the data collected in different sea areas are different, which is helpful to verify the generalization ability of MRF-Net. In the detection part, we detected the object—fishnet—in real time with an embedded single board computer (SBC) MIO2361 whose processor is Intel^®^ Atom^TM^ E3900 series and the NVIDIA Jetson AGX Xavier embedded system module of an AUV platform in our lab. Then, we sent the detection results, including the object position and angle information, to the control module of the AUV via the user datagram protocol (UDP) protocol. The control module can use the detection results to avoid fishing nets. As this paper focuses on fishing net detection, the details of the obstacle avoidance algorithm are not discussed here. However, the results of the fishing net avoidance experiment verify the effectiveness of our proposed method. Figure 14 shows the structure of our AUV platform and the scenes of our fishing net avoidance experiment. In order to ensure the UDP communication between devices, we used a switch to place the devices in the same local area network (LAN). The connection between these devices is shown in Figure 15.

**Figure 16:**
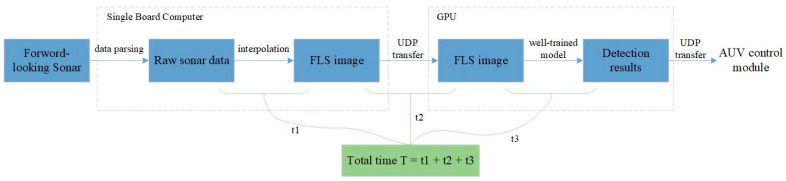
The process of the real-time detection experiment. Note: t1 is the time of interpolation, t2 is the time of transferring a FLS image, and t3 is the inference time of the network.

The experimental process is shown in Figure 16. In our system, the SBC receives four frames of original data per second from forward-looking sonar. When the scanning range is set to 15 m, each beam returns 1875 pixels. That is to say, SBC can get 1875 × 256 original data matrix after data parsing. In order to reduce the transmission time, we uniformly sampled the original data to obtain a 430 × 256 data matrix and then interpolated it to 430 × 744. As shown in Figure 16, in the real-time experiment, the time t1 for interpolating was 26 ms; t2, which represents the time taken to transfer a FLS image from the SBC module to GPU, was 0.104 ms. Then, the image was fed into the well-trained model to obtain the detection results, which were finally sent to the control module of the AUV. As shown in Table 4, the inference time t3 of MRF-Net was 89 ms. Therefore, the total processing time of a FLS image for MRF-Net is 0.219 ms, which means that the object detection of a FLS image takes less time than the time interval for receiving a new FLS image to process.

**Figure 17:**
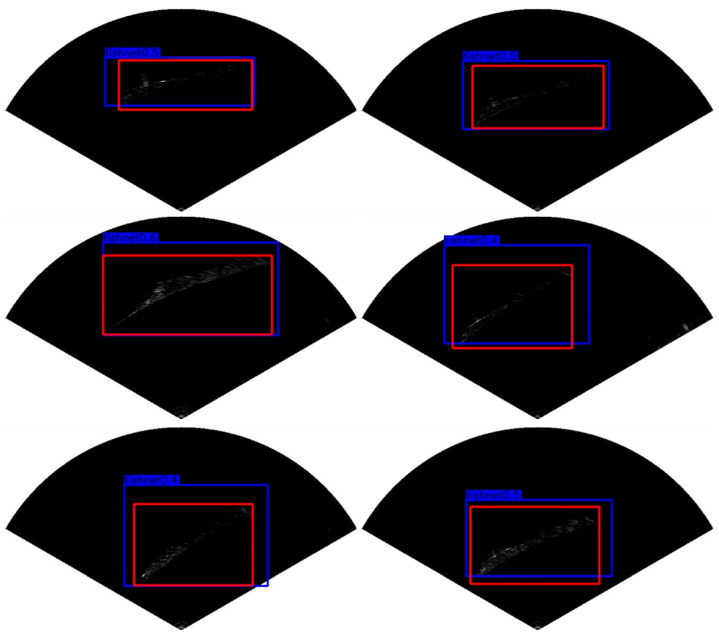
The real-time results of detecting fishing nets with our proposed MRF-Net. Note: the red box is the ground truth of the manual marker, the blue box is the detection result of MRF-Net.

We compared the saved real-time detection results with the ground-truth of offline manual annotation. The results are shown in Figure 17. We collected 5184 FLS images as a testing set in the experiment to measure the generalization ability of MRF-Net. In terms of mAP, our results show that MRFNet can achieve a mAP of 87.3%, which is comparable to the performance in Table 3.

**Figure 18:**
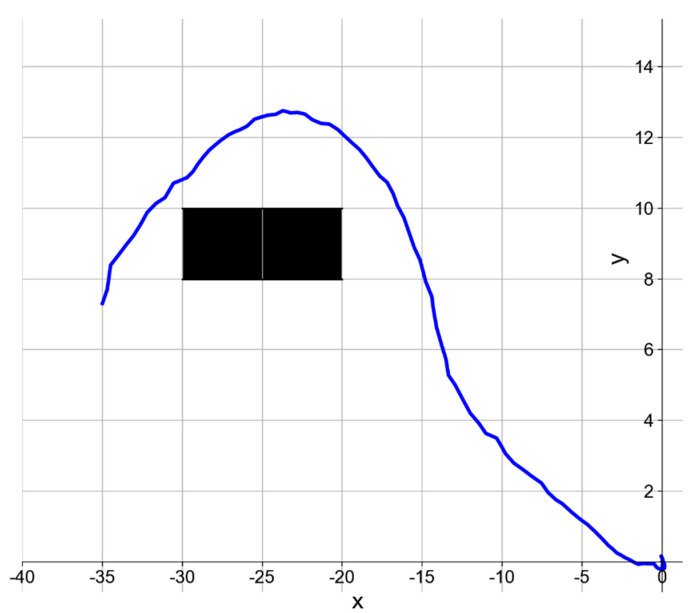
The trajectory of AUV in the fishing net avoidance experiment. The blue line rep-resents the navigation trajectory of the AUV, and the grid filled with black represents the position of the fishing net.

In addition, in the fishing net avoidance experiment, the AUV carried out a fixed-point straight-line path following task. That is to say, the starting and ending points of the straight-line path were randomly selected to place the fishing net on the path. The speed of the AUV was 3 knots. If the fishing net is detected, the AUV is expected to turn to avoid it and then return to the original path. Figure 18 shows the navigation path of the AUV when avoiding the fishing net in the experiment. We carried out the fishing net avoidance experiment 16 times, and the AUV successfully avoided it 13 times. After carefully checking, we found that the reason for failing to avoid the fishing net in those three trials is that there were too few detected sonar images of the fishing net because of the high confidence threshold set to reduce false detections. This is insufficient to guide the AUV to take the correct avoidance action. However, our results show that MRF-net can complete the real-time detection task, and the accuracy of the detection results is sufficient to support the subsequent AUV obstacle avoidance task.

In summary, our results show that MRF-Net has a good generalization ability and can meet the requirements of real-time detection tasks in terms of speed and accuracy using FLS images.

## 6. Conclusions

In this paper we have presented a novel architecture based on a deep convolutional neural network designed for fishing net detection of an AUV using forward-looking sonar. The architecture is designed with an encoder-decoder structure consisted of the multi-branch block, which can learn rich features. Moreover, we used the mixup strategy in our training process to improve the accuracy of detection and the generalization ability of the network. We trained and tested our network on the data collected in the sea by comparing it with some of the most popular object detection algorithms. We also evaluated our network by conducting a real-time experiment detecting fishing nets in a different sea area with the NVIDIA Jetson AGX Xavier of our AUV platform. The experimental results show that in terms of computational complexity, inference time and prediction accuracy, MRF-Net can meet the requirements of real-time detection and obstacle avoidance tasks of the AUV. At present, our research mainly focuses on fishing net detection. In future work, we inten to improve the detection accuracy and extend our method to detect and classify multiple obstacles at the same time.

## Figures and Tables

**Table 1:** Technical parameters of Gemini 720i.

Technical Parameter	
Operating frequency	720 kHz
Voltage supply	20–75 V DC
Detection range	0.2–120 m
Number of beams	256
Visual angle	120°
Angular resolution	0.5°
Range resolution	8 mm
Vertical beam width	20°
Size	228 mm × 135 mm × 110 mm

**Table 2:** Definitions of indicators.

Symbol	Determinant
TP	Number of predicted bounding boxes whose IoU with the ground truth are higher than 0.5
FP	Number of predicted bounding boxes whose IoU with the ground truth are less than or equal to 0.5
TN	Not used
FN	Number of undetected ground truth

**Table 3:** Comparison of prediction accuracy on our collected dataset (the best result is bolded).

Method	mAP (%)	Cloth (%)	Fishnet (%)	Plastic Bag (%)
CenterNet-dla [10]	90.0	86.3	93.0	90.7
Faster RCNN [16]	86.7	79.7	92.3	88.2
YoloV3 [21]	71.2	42.0	83.6	87.9
RFBNet [25]	87.2	83.3	87.3	**90.9**
SSD300 [33]	80.6	71.4	**88.1**	82.4
MRFNet(Ours)	**90.3**	**88.2**	91.8	90.7

**Table 4:** Comparison of networks in terms of number of parameters, model size, the amount of calculation (GFLOPs), and frames per second (FPS) (the best result is bolded).

Method	Parameters	Model Size	GFLOPs	Time(ms)	FPS
CenterNet-dla [10]	17.9 M	70.5 M	30.9 G	127	7.87
Faster RCNN [16]	417.9 M	547.0 M	231.3 G	957	1.04
YoloV3 [21]	61.9 M	246.4 M	33.0 G	94	10.64
RFBNet [25]	34.1 M	136.6 M	35.0 G	99	10.01
SSD300 [33]	23.7 M	96.1 M	30.4 G	113	8.85
MRF-Net (Ours)	**6.4 M**	**26.1 M**	**18.5 G**	**89**	**11.13**

**Table 5:** Effectiveness of techniques in our proposed MRF-Net.

							MRF-Net
Median filter			√				
Lee filter				√			
Our preprocessing method					√	√	√
IBN layer						√	√
Dilated convolution		√	√	√	√	√	√
Mixup strategy							√
mAP	87.6	88.6	88.3	88.1	89.2	89.7	**90.3**

## Data Availability

Because the data involves privacy, and the project used to support the paper is a classified military project, the data cannot be available publicly.
